# Cost associated with a relapse-free patient in multiple sclerosis: A real-world health indicator

**DOI:** 10.1371/journal.pone.0267504

**Published:** 2022-04-29

**Authors:** Lucía Romero-Pinel, Laura Bau, Elisabet Matas, Isabel León, Roser Juvany, Ramon Jódar, Antonio Martínez-Yélamos, Sergio Martínez-Yélamos

**Affiliations:** 1 Multiple Sclerosis Unit, Department of Neurology, Hospital Universitari de Bellvitge–Institut d’Investigació Biomèdica de Bellvitge, L’Hospitalet de Llobregat, Barcelona, Spain; 2 Department of Pharmacy, Hospital Universitari de Bellvitge–Institut d’Investigació Biomèdica de Bellvitge, L’Hospitalet de Llobregat, Barcelona, Spain; 3 Departament de Ciències Clíniques, Facultat de Medicina, Universitat de Barcelona, Barcelona, Spain; Universita degli Studi di Napoli Federico II, ITALY

## Abstract

**Background:**

The efficacy and safety of disease-modifying therapies (DMTs) in multiple sclerosis (MS) are well known; however, owing to their high costs, determining real-world outcomes is essential to evaluate the cost-effectiveness of different therapeutic strategies. This study aimed to investigate the variability in the annual cost of DMTs associated with a relapse-free patient in a representative population cohort of relapsing-remitting MS (RRMS), and whether this could serve as an appropriate health indicator.

**Methods:**

We analyzed the patients followed up in our MS clinic during the years 2016 and 2019, and selected patients belonging to our health district diagnosed with RRMS. The treatment cost associated with a relapse-free patient was the ratio between the total cost of DMTs and the number of relapse-free patients, treated and not treated, during the year of the study.

**Results:**

A total of 158 patients with RRMS in 2016 and 183 in 2019 were included in our study. In 2016, 101 patients with RRMS (63.9%) received treatment with DMTs and 120 patients (75.9%) remained relapse-free. The mean cost of DMTs per patient in 2016 was €7414.3 (95% confidence interval [CI]: 6325.2–8503.4) considering all the patients (treated and not treated). In 2019, 126 patients (68.9%) received DMTs and 151 patients (82.5%) remained relapse-free. The mean cost of DMTs per patient in 2019 was €6985.4 (95% CI: 5986.9–7983.9) considering all the patients. The cost per year of DMTs to achieve a relapse-free patient was €9762.2 in 2016 and €8465.8 in 2019.

**Conclusions:**

The treatment cost per year to achieve a relapse-free patient was stable during successive measurements in the same population. Therefore, it may be considered a good real-world health indicator for patients with RRMS treated with DMTs.

## Introduction

Multiple sclerosis (MS) is a chronic autoimmune demyelinating disease of the central nervous system, which is the most frequent non-traumatic cause of disability among young adults [1]. Therefore, it results in a significant loss in the quality of life of patients as well as their families and social network, with a consequent huge socioeconomic impact [2].

The cost-of-illness studies conducted in recent years to evaluate the economic burden of MS [3] have demonstrated a good correlation with disease severity, since higher costs have been observed in more disabled patients [4, 5]. Although a significant percentage of the economic burden in these patients is owing to indirect costs, such as work disability [6], the cost resulting from the use of disease-modifying therapies (DMTs) has risen significantly over the past two decades. These pharmacological costs constitute the main expenditure in patients with mild disability as well as in MS overall. In a study conducted in our MS unit, DMTs accounted for more than half of the direct costs in the mild and moderate stages of the disease [7].

Early interventions for MS are of utmost importance in reducing future disability. Thus, when treating patients, we aim to reduce the severity of the disease in the advanced stages, which could subsequently reduce the total economic burden of MS. Cost-effectiveness studies are usually designed mainly to evaluate specific interventions rather than different clinical management strategies. Owing to the increased cost of DMTs and limited available resources to healthcare systems, it is essential to assess health indicators in relation to the cost-effectiveness of therapeutic strategies in a real-world setting. A health indicator is a measure that, usually employ a ratio that provides comparable information across different populations and/or over time [8]. In our study, we propose a new health indicator, including not only treated patients but also untreated patients, in the analysis. Including untreated patients in the healthcare outcome could help evaluate the effect of over- or under-treatment upon comparing the results among different healthcare providers. Thus, the concomitant costs of relapse-free patients could be employed as an index of cost-effectiveness.

This study aimed to investigate the variability in the annual cost of DMTs associated with a relapse-free patient, and whether this, could be an appropriate health indicator for evaluating the cost-effectiveness of different therapeutic strategies in relapsing-remitting MS (RRMS).

## Patients and methods

### Study population

This study included patients recruited from the MS clinic of the Hospital Universitari de Bellvitge, which is the only center for demyelinating disease in our health district. This district comprised 201192 inhabitants on December 31, 2016 [9] and 203779 on December 31, 2018, which are the most recently published data [10]. Our center is located in Barcelona, Catalonia, northeast Spain. For the present study, patients who visited our MS clinic during 2016 and/or 2019, diagnosed with MS according to the McDonald criteria [11, 12], and belonging to our health district were evaluated. Patients diagnosed with RRMS were selected for the analysis [13]. Patients were classified into two cohorts, the *2016* and *2019 cohorts*. Some patients were eligible for inclusion in both the cohorts.

This study was approved by the Hospital Universitari de Bellvitge Research Ethics Committee (PR356/21). Patients signed informed consent forms, and data were collected anonymously.

### Clinical outcomes

The clinical data for both cohorts, 2016 and 2019, were prospectively collected in a real-world setting using the European Database for Multiple Sclerosis (EDMUS) [14]. All the patients were examined by a qualified neurologist following routine clinical examination at least once every six months and at the time of relapse. Disease severity was assessed using the Expanded Disease Status Scale (EDSS) [15] by a certified neurologist (neurostatus.net) [16]. Clinical outcomes collected in our database included the presence of relapses and worsening of disability as measured by the EDSS score. Relapse was defined as a new neurological symptom lasting at least 24 hours accompanied by neurological signs and with the absence of fever or infection. Worsening of disability was defined as a 1.0-point increase in the confirmed EDSS score at 6 months. Magnetic resonance imaging (MRI) data were retrospectively retrieved from the clinical records of the patients who had undergone routine MRI. All the MRIs scans were previously evaluated by a qualified neuroradiologist considering new T2 lesions or gadolinium-enhancing lesions.

The proportion of patients with no evidence of disease activity (NEDA-3) was analyzed using the MRI data. The NEDA-3 definition used for this study included no relapses, no evidence of a 1.0-point increase in the confirmed EDSS score at 6 months, and no new T2 or gadolinium-enhancing lesions on MRI. NEDA-3 was analyzed during the years studied (January to December 2016 and 2019).

### Economic outcomes

The cost of the DMTs for each patient was provided by the Department of Pharmacy of our center using an anonymized database and was based on the hospital’s acquisition price.

Patients from each cohort were classified into two groups: treated and not treated with DMTs. The criteria established by the Catalan Health Service (Catsalut), which finances healthcare in Catalonia, were used for treating the patients. Treated and untreated patients were further classified into two groups according to the presence or absence of at least one relapse during the respective years of analysis.

The total cost of DMTs includes the pharmacological costs of all the patients with RRMS, relapsing patients, and relapse-free patients. The treatment cost associated with a relapse-free patient was presented as an index calculated as the ratio between the total cost of the DMTs and the number of relapse-free patients in the entire cohort, treated and untreated patients, during each year of the study. The same analysis was performed with the data obtained during 2016 and 2019 to evaluate the variability of the index between the two close points in time. The analysis was performed on the same population under the same clinical circumstances.

### Statistical analysis

Statistical analysis was performed to compare the clinical characteristics of the patients and analyze the costs of DMTs during 2016 and 2019 for the patients with RRMS and in the different patient groups. The mean and standard deviation (SD), median and range, and percentage (%) are used to describe the population characteristics. The mean, SD and 95% confidence intervals (CIs) are used to describe the costs of the DMTs. We performed a sensitivity analysis of the proposed health indicator by calculating the same index for the values of the 95% CI of the relapse-free patient proportion in each year. For the univariate analysis, the Pearson chi-squared test, Student’s test and Mann Whitney *U* test were performed accordingly. SPSS Statistics for Windows version 20 was used for statistical analysis. A difference of p<0.05 was considered statistically significant for each comparison.

## Results

### 2016 cohort

A total of 181 patients diagnosed with MS in our health district were observed in our MS unit during 2016. The hospital-based prevalence of MS is 89.9 per 100 000 inhabitants. Among these, 158 patients diagnosed with RRMS were included in our study. The patients’ baseline characteristics are shown in Table 1. One hundred and one patients (63.9% of the cohort, 95% CI: 56.2%–71.0%) were treated with DMTs. A description of the DMTs used in 2016 is provided in Table 2. The number of DMTs did not correspond to the number of treated patients since 12 patients used two different DMTs in 2016. One hundred and twenty patients (75.9% of the cohort, 95% CI: 69.3%–82.6%) remained free of relapses during the year, considering the treated and untreated patients. The disease characteristics and cost of treatment in the different patient groups of the 2016 cohort are shown in Table 3. MRI data were not analyzed in 2016, as fewer than 50% of the patients underwent MRI during the year. Consequently, NEDA-3 was not analyzed in 2016.

**Table 1:** Baseline characteristics of RRMS from the 2016 and 2019 cohorts.

	RRMS 2016 cohort n = 158	RRMS 2019 cohort n = 183	p-value
**Female, n (%)**	112 (70.9)	124 (67.8)	0.533
**Age at onset (years), mean (SD)**	30.6 (10.9)	31.8 (10.8)	0.323
**Disease duration (years), mean (SD)**	16.8 (10.8)	16.3 (11.7)	0.659
**EDSS, median (range)**	2 (0–7.5)	2 (0–7.5)	0.775

RRMS, relapsing-remitting multiple sclerosis; EDSS, Expanded Disability Status Scale.

**Table 2:** DMTs used by the patients with RRMS from the 2016 and 2019 cohorts.

	DMTs RRMS 2016 cohort (n)	2016 Annual cost (euros)	DMTs RRMS 2019 cohort (n)	2019 Annual cost (euros)
**Interferon beta**	66	578 961	46	345 705
**Glatiramer acetate**	14	91 154	20	63 700
**Teriflunomide**	-	-	8	58 000
**Dimethyl fumarate**	5	26 845	12	96 948
**Fingolimod**	11	190 982	20	337 040
**Natalizumab**	15	237 645	15	197 085
**Ocrelizumab**	-	-	11	109 615
**Rituximab**	-	-	4	10 128
**Alemtuzumab**	2	45 878	1	18 828
**Cladribine**	-	-	2	41 288

DMTs, disease-modifying treatments; RRMS, relapsing-remitting multiple sclerosis; n, number of patients treated with DMTs

**Table 3:** Disease characteristics of the 2016 cohort and cost of the DMTs.

	Treated (n = 101)	Not treated (n = 57)	p-value	Relapse free (n = 120)	≥1 relapse (n = 38)	p-value
**Female, n (%)**	76 (75.2%)	36 (63.2%)	0.108	88 (73.3%)	24 (63.2%)	0.229
**Age at onset (years), mean (SD)**	30.0 (10.6)	31.9 (11.2)	0.295	31.33 (10.9)	28.5 (10.4)	0.162
**Disease duration (years), mean (SD)**	15.2 (10.0)	19.8 (11.7)	0.010	17.9 (11.2)	11.5 (7.7)	<0.001
**EDSS, median (range)**	2 (0–7.0)	1.5 (0–7.5)	0.004	2 (0–7.5)	2 (0–7)	0.649
**Relapse-free patients, n (%)**	68 (67.3%)	52 (91.2%)	<0.001	--	--	--
**Treated patients, n (%)**	--	--	--	68 (56.7%)	33 (86.8%)	<0.001
**Cost of treatment (€, euros), mean (SD)**	11598.6 (5137.9)	--	--	6362.2 (6333.8)	10736.71 (7738)	0.001

DMTs, disease-modifying treatments; SD, standard deviation; p, significance level; EDSS, Expanded Disability Status Scale.

The mean cost of the DMTs per patient in 2016 was €7414.3 (95% CI: 6325.2–8503.4) upon considering when we considered all the patients with RRMS, treated and untreated. The cost of the patients who had a relapse was greater than the cost of patients who were free of relapses during 2016. The annual cost of the DMTs to achieve a relapse-free patient was €9762.2, considering treated and untreated patients. This amount is the result of dividing the total pharmacy cost (1 171 465 euros) by 120, which was the number of relapse-free patients in 2016.

2016 Annual cost of DMTs associated with a relapse-free patient (€)

= Total cost of DMTs (€) / number of relapse-free patients

= €1 171 465 euros / 120 = €9762.2

### 2019 cohort

A total of 214 patients belonging to our health district, diagnosed with MS, and who visited our MS unit in 2019, were selected. The hospital-based prevalence of MS is 105 patients per 100 000 inhabitants. Among the 214 patients, 183 patients had RRMS, who were included in our study. The baseline characteristics are shown in Table 1.

One hundred and twenty-six patients (68.9% of the cohort, 95% CI: 62.1%–75.6%) were treated with DMTs during 2019. A description of the DMTs used in 2019 is provided in Table 2. The number of DMTs did not correspond to the number of treated patients since 13 patients used two different DMTs in 2019. One hundred and fifty-one patients (82.5% of the cohort, 95% CI: 77%–88%) remained relapse-free during 2019. One hundred and seventy-one patients did not show an increase in their EDSS (93.4%) in 2019. A total of 144 patients (78.7%) underwent MRI in 2019; 126 of these (87.5%) had no new T2 lesions or gadolinium-enhancing lesions; the proportion of patients with NEDA-3 in these 144 patients was 69.4%.

The disease characteristics and cost of treatment in the different patient groups of the 2019 cohort are shown in Table 4.

**Table 4:** Disease characteristics of the 2019 cohort and cost of the DMTs.

	Treated (n = 126)	Not treated (n = 57)	p-value	Relapse free (n = 151)	≥1 relapse (n = 32)	p-value
**Female, n (%)**	89 (70.6%)	35 (61.4%)	0.216	103 (68.2%)	21 (65.6%)	0.776
**Age at onset (years), mean (SD)**	31.5(10.9)	32.35 (10.4)	0.652	32.1 (10.2)	30.3 (13.3)	0.01
**Disease duration (years), mean (SD)**	14.4 (10.4)	20.5 (13.2)	0.003	17.2 (11.8)	12.1 (10.0)	0.025
**EDSS, median (range)**	2 (0–7.5)	1.5 (0–7.5)	<0.001	2 (0–7.5)	2.5 (0–5.5)	0.030
**Relapse-free patients, n (%)**	99 (78.6%)	52 (91.2%)	<0.05	--	--	--
**Treated patients, n (%)**	--	--	--	99 (65.6%)	27 (84.4%)	<0.05
**Cost of DMTs (€, euros), mean (SD)**	10145.6 (5993.0)	--	--	6454.9 (6521.2)	9489.0 (7843.5)	0.022

DMTs, Disease modifying treatments; SD, standard deviation; p, significance level; EDSS, Expanded Disability Status Scale.

The mean cost of DMTs per patient in 2019 was €6985.4 (95% CI: 5986.9–7983.9) when we considered all patients, relapse-free and relapsing patients as well as treated and not treated patients. The cost of patients who have had a relapse were greater than the cost of patients free of relapses during this year. The annual cost of DMTs associated with a relapse-free patient was €8465.8 euros in 2019.

2019 Annual cost of DMTs associated with a relapse-free patient (€)

= Total cost of DMTs (€) / number of relapse-free patients

= €1 278 337 euros / 151 = €8465.8 euros

### Comparison of the 2016 and 2019 cohorts

We compared the 2016 and 2019 cohorts that comprised 158 and 183 RRMS patients, respectively. From them, 139 patients were elegible for both cohorts. No differences were found upon comparing the baseline characteristics of both the cohorts (sex, age at onset, disease duration or EDSS). A higher proportion of patients were treated with DMTs in 2019 than in 2016, although this did not reach significance (68.9% vs. 63.9%, p = 0.336). No significant difference was observed between the proportions of relapse-free patients (75.9% in 2016 vs. 82.5% in 2019, p = 0.135). The mean cost of the DMTs considering all the patients with RRMS was lower in 2019; however, the result was not significant (€6985.4 [95% CI: 5986.9–7983.9] in 2019 versus €7414.3 [95% CI: 6325.2–8503.4] in 2016, p = 0.156). The annual cost incurred to achieve a relapse-free patient was €9762.2 in 2016 and €8465.8 in 2019. The variability between 2016 and 2019 was 13.28%. The sensitivity analysis of the proposed health indicator was €8976.06–€10699.29 in 2016 and €7938.01–€9072.01 in 2019.

## Discussion

We describe a new health indicator, which is the treatment cost associated with a relapse-free patient, in the real world of MS. This outcome is the ratio between the annual pharmacological cost of the DMTs for the entire population and the number of patients who remained relapse-free each year. In our study, the treatment cost to obtain a relapse-free patient was similar in the 2016 and 2019 cohorts at €9762.2 and €8465.8, respectively. Therefore, we suggest that the proposed outcome may be consistent over time, which is of utmost importance concerning outcome reliability [17]. This indicator increases when the patients are overtreated. In this case, the total cost of the DMTs would increase with no effect on the number of relapse-free patients. However, undertreating patients would not cause the new indicator to decrease since we expected an increase in the number of relapsing patients.

The clinical characteristics of the patients were analyzed to evaluate whether the 2016 and 2019 cohorts were comparable. We found no differences in the clinical characteristics of the cohorts, which were similar to other published cohorts in the treatment era [18]. We observed a longer disease duration and lower EDSS score in the untreated patients, probably owing to the so-called benign MS that may be found in the MS population-based cohorts at different prevalence rates [19]. The variability in the new health indicator between 2016 and 2019 in our cohort was approximately 13%. We assumed this variability to be acceptable, since the sensitivity analysis demonstrated an overlap of the indexes. The comparison was performed within the same population at two close time points, and with the same therapeutic strategy in both the cohorts.

Hence, this new health indicator can be used to monitor the adequacy of the treatment management strategies, such as the well-known dilemma of induction versus escalation [20]. Recent studies comparing Danish and Swedish national treatment strategies, using more efficacious DMT as initial treatment, demonstrated a higher reduction in worsening of disability in Sweden [21]. Considering this setting, it would be interesting to evaluate the costs of both the strategies, and estimate whether they are proportional to efficacy. The indicator we proposed could help compare the treatment strategies. This new indicator possesses the strength of taking into account untreated patients, avoiding bias between strategies when untreated patients are not considered. This indicator would also be useful for comparing several healthcare providers during a benchmarking process or for monitoring the effectiveness of the same healthcare provider over time.

The efficacy of DMTs in the treatment of MS is well known. National health systems could also use the proposed indicator to evaluate real-world effectiveness and establish the prices of new drugs. Pharmacological innovations may improve the effectiveness of treatment but with a disproportionate cost for the healthcare system sometimes. We can assume the effectiveness of a new drug if it has a higher pharmacological cost than a previous one but it proportionally increases the number of relapse-free patients.

At the same time, with scarce resources and rising costs of DMTs [22], it is crucial to implement risk-sharing payment schemes [23]. Studies of treatment effectiveness in routine clinical practice are limited but are essential to identify meaningful outcomes in this setting [24]. The proposed cost outcome can help implement the aforementioned schemes [25]. The use of risk-sharing agreements is gradually becoming established, while at the same time, is believed to favor the introduction of personalized medicine [26]. An example of a risk-sharing scheme in MS that has already been introduced involves the use of β-interferon and glatiramer acetate by the UK National Health Service [27]. Recently, in our area, Catsalut performance-based risk-sharing agreements [28] have established oncological treatments, as published in other populations [29]. It is worth noting that one of the performance-linked risk agreements in Catalonia signed between Catsalut and a pharmaceutical company is for fampridine, which is used in patients with MS and gait disability.

The strength of our study was that it was a population-based study. The hospital-based prevalence of MS observed in 2016 and 2019 was representative of the epidemiological population-based studies performed in our area. A study carried out in Catalonia demonstrated that the crude prevalence of MS was 79.9 (95% CI: 66.3–95.6) per 100 000 inhabitants [30], while in another recently published paper from southeast Spain, the non-adjusted prevalence of MS was 111.9 (95% CI: 87.7–142.9) cases per 100.000 inhabitants [31]. Considering these results, we can assume that our hospital-based prevalence was very similar to our expected population-based prevalence.

Our study had certain limitations. First, it was conducted in only one center belonging to a public healthcare system, and thus may not be reproducible in other healthcare systems. Second, there was a lack of NEDA-3 analysis in 2016. We evaluated NEDA-3 in a subgroup of the 2019 cohort, but not in 2016, owing to the limited number of patient imaging procedures performed. Nevertheless, this fact made us aware that NEDA-3 was not the best health outcome for our study, since it could be conditioned by the patients who underwent MRI. Patients with greater disease activity underwent more imaging tests. Despite this, our analysis demonstrated a higher percentage of patients with NEDA-3 compared to other published real-world cohorts [32, 33]. Our cohort may have been more benign owing to its similarity to a population-based cohort. Notably, NEDA-4 was able to predict MS outcomes better than NEDA-3 [34]. However, we were unable to analyze NEDA-4 since the changes in brain volume were not routinely evaluated.

Thus, we were able to address the need to find meaningful and easily measurable result-based health outcomes [25]. In some studies, cost-based indicators were the focus of research; however, they were not implemented in clinical practice [35]. Our study demonstrated that the cost associated with a patient with relapse-free MS for one year is consistent over time. Future studies should compare our results with other MS population-based cohorts to analyze their reproducibility.

## Conclusions

In conclusion, the annual treatment cost associated with a relapse-free patient could be a new health indicator considering its stability in successive measurements in the same population. Thus, it may be considered a good health indicator for patients with DMTs to help healthcare decision-makers allocate limited resources.
